# Determinants of Sensitivity to Radiotherapy in Endometrial Cancer

**DOI:** 10.3390/cancers12071906

**Published:** 2020-07-15

**Authors:** Maria Alba Sorolla, Eva Parisi, Anabel Sorolla

**Affiliations:** 1 Research Group of Cancer Biomarkers, Biomedical Research Institute (IRB Lleida), 25198 Lleida, Spain; msorolla@irblleida.cat (M.A.S.); eparisi@irblleida.cat (E.P.); 2 Harry Perkins Institute of Medical Research, QEII Medical Centre, Nedlands and Centre for Medical Research, The University of Western Australia, Crawley, Western Australia 6009, Australia

**Keywords:** endometrial cancer, radiotherapy, signaling pathways, clinical trials, radiogenomics

## Abstract

Radiotherapy is one of the cornerstone treatments for endometrial cancer and has successfully diminished the risk of local recurrences after surgery. However, a considerable percentage of patients suffers tumor relapse due to radioresistance mechanisms. Knowledge about the molecular determinants that confer radioresistance or radiosensitivity in endometrial cancer is still partial, as opposed to other cancers. In this review, we have highlighted different central cellular signaling pathways and processes that are known to modulate response to radiotherapy in endometrial cancer such as PI3K/AKT, MAPK and NF-κB pathways, growth factor receptor signaling, DNA damage repair mechanisms and the immune system. Moreover, we have listed different clinical trials employing targeted therapies against some of the aforementioned signaling pathways and members with radiotherapy. Finally, we have identified the latest advances in radiotherapy that have started being utilized in endometrial cancer, which include modern radiotherapy and radiogenomics. New molecular and genetic studies in association with the analysis of radiation responses in endometrial cancer will assist clinicians in taking suitable decisions for each individual patient and pave the path for personalized radiotherapy.

## 1. Introduction

Endometrial cancer (EC) was the second most frequent gynecologic malignancy and the sixth most diagnosed cancer in women worldwide in 2018 [1]. Around 75% of ECs are confined in the uterine corpus and 15–20% recur after primary surgery. Of them, approximately 1/3 recurs in the vagina and pelvic regions and 2/3 at distant sites [2]. Patients with localized EC present a 5-year survival rate of 95% and such a rate decreases to 16% with the presence of distant metastasis [3].

The vast majority of ECs are adenocarcinomas. They have been classically divided in two different histological types: type 1 or endometrioid carcinomas, and type 2, which include uterine serous carcinomas and clear cell carcinomas [4]. Type 1 carcinomas account for 70–80% of all ECs. They are low-grade moderate to well differentiated tumors and have good prognosis. In contrast, type 2 tumors, which account for 10–20% of all ECs, are high-grade poorly differentiated tumors and have poor prognosis due to their high risk of recurrence and metastasis [4]. The genomic sequencing initiative ’The Cancer Genome Atlas’ has allowed the identification of four different molecular subtypes of ECs: polymerase ε exonuclease (POLE) ultramutated, microsatellite instability (MSI) hypermutated, copy-number low and copy-number high [5]. When comparing with the traditional classification, the authors found that 40% of the endometrioid tumors are MSI while MSI is present in only 2% of serous tumors.

Pathological stage plays a crucial role in treatment decision of ECs. Current forms of radiotherapy (RT) to treat ECs consist of high-energy external beam radiation therapy (EBRT) and brachytherapy (BT), the placement of radioactive isotopes into the vagina. Although RT has been successful in preventing local recurrences after surgery, there is still risk of RT failure with patterns of local and distant recurrences; 5-year probability of distant recurrences of 29.1% in patients with high-risk EC treated with RT alone has been reported [6]. Therefore, research focused on finding the molecular mechanisms that determine the acquisition of resistance and a lack of sensitivity to RT and consequent cancer relapse in EC is essential.

In the current work, we have reviewed the molecular determinants of RT sensitivity in EC, which include the status of the cancer survival pathways phosphatidylinositol 3-kinase (PI3K)/PTEN/protein kinase B (AKT)/mammalian target of rapamycin (mTOR), nuclear factor-kappa B (NF-κB) and mitogen-activated protein kinase (MAPK) pathway; oncoproteins such as tyrosine kinase receptors and growth hormone; proteins involved in DNA repair mechanisms and the immune system. Additionally, we have listed the current clinical trials studying the effects of targeted therapies directed to the above cellular mechanisms with RT in EC. Furthermore, we have provided an overview of the latest cutting-edge advances in RT that have the potential to lead the development of targeted RT for EC.

## 2. Current State of the Art in Treatment of Endometrial Cancer

The most established treatment options for EC are surgery, RT and standard chemotherapy. There are also two approved targeted therapies for clinical use, which are megestrol acetate [7] and the combination of pembrolizumab and lenvatinib [8].

The selection of treatment is done after rigorous examination, which considers the following factors: family history, patients’ performance status, diagnostic features, clinical staging and risk assessment. Clinical staging, I-IV must follow The International Federation of Gynecology and Obstetrics (FIGO) classification, first described in 1988 [9] and revised later in 2009 [10]. Risk assessment is based on clinicopathological prognostic factors, which include age, FIGO stage, depth of myometrial invasion, tumor differentiation grade, tumor type (endometrioid versus serous and clear cell) and lymphovascular space invasion.

The clinical practice guidelines of the European Society of Medical Oncology (ESMO) for the management of EC patients constitutes an important tool for the application of the suitable treatment [11]. According to ESMO, early-stage low-risk EC patients should be managed with surgery alone, in particular total hysterectomy and bilateral salpingo-oophorectomy. Intermediate-risk EC could be considered for lymphadenectomy for staging, and adjuvant BT is recommended for treatment. Intermediate-high risk EC patients should be treated with adjuvant BT or EBRT, depending on the nodal status and histological grade. High-risk EC patients could be recommended for lymphadenectomy and adjuvant EBRT, combined with chemotherapy under specific circumstances. This in agreement with the results of the GOG-249 trial [12], which demonstrates that RT alone remains an effective, well-tolerated and appropriate adjuvant treatment in high-intermediate and high-risk early-stage EC. Stage II patients can undergo radical hysterectomy. Lymphadenectomy is recommended to guide staging and adjuvant therapy. In this stage, adjuvant therapy will include vaginal BT, EBRT and BT boosts. They can be combined with chemotherapy, depending on nodal staging and tumor grade. In stage III patients, surgery will include complete macroscopic cytoreduction. Adjuvant chemotherapy plus EBRT will be considered in IIIA, IIIB and IIIC1 stages or plus extended field EBRT in the IIIC2 stage. In high-risk non-endometrioid cancer, the adjuvant options are vaginal BT or EBRT plus chemotherapy depending on stage and histology. In these cases, chemotherapy is highly encouraged through clinical trials. However, chemotherapy seems to only elicit a modest effect in avoiding recurrence in high risk and/or advanced disease. The administration of RT with concurrent adjuvant chemotherapy did not improve progression-free survival (PFS) and overall survival (OS) in the GOG-258 trial with 813 cases of stage III-IV EC, but significantly decreased vaginal and para-aortic recurrences compared to chemotherapy alone [13]. Similarly, results from the PORTEC-3 clinical trial suggested that adjuvant chemotherapy given during and after RT for high-risk endometrial cancer did not improve 5-year OS but did increase failure-free survival across several risk histologies [14]. Finally, in metastatic disease (stage IV), palliative surgery can be performed to alleviate specific symptoms. Additionally, radical RT (intrauterine BT ± EBRT) or palliative RT should be taken in account to ameliorate patient’s pain related to local recurrence or systemic disease.

## 3. Signaling Pathways and Cellular Processes Modulating Radiotherapy Response in Endometrial Cancer and Preclinical Investigations

### 3.1. PI3K/PTEN/AKT/mTOR Signaling Pathway

The PI3K/PTEN/AKT/mTOR signaling pathway is aberrantly activated in 80% of endometrioid ECs mostly due to PTEN loss [15]. Other molecular events are the amplification and mutations in phosphoinositide-3-kinase catalytic subunit alpha (*PIK3CA*) [16], mutations in *PIK3 regulatory subunit 1* (*R1*) and *PIK3KR2* [17]. Mutations in *AKT*, in particular *AKT1*, are also present but infrequent, affecting 2% of type 1 ECs [18].

It has been shown that ionizing radiation activates PI3K/AKT/mTOR pathway in EC [19,20], suggesting that its inhibition could elicit radiosensitization effects. Indeed, pharmaceutical inhibition of PI3K/mTOR by the dual PI3K/mTOR inhibitor NVP-BEZ235 sensitized five EC cell lines to RT [20]. Additionally, our group demonstrated that the inhibition of AKT by Sunitinib sensitized four EC cells lines to ionizing radiation [19], occurring only when total AKT inhibition was achieved [19]. In contrast, a study shows that most of the EC cell lines carrying oncogenic mutations in *PIK3CA* coexisting with *PTEN* mutations and with observed AKT activity were sensitive to RT [21].

Regarding PTEN, Mukherjee et al. demonstrated that PTEN improves DNA repair and prevents cell death in the EC Ishikawa cells transduced with PTEN or PTEN able to translocate to the nucleus when treated with the PARP inhibitor Olaparib [22]. For this reason, it is believed that PTEN loss could fail to provide effective DNA damage response after the double-strand breaks (DSBs) induced by RT. In fact, PTEN restoration renders Ishikawa cells more resistant to RT [19,22]. Additionally, RL95-2 cells (PTEN null) are more sensitive to RT than KLE cells (wild type) [19]. Overall, it seems that PTEN status predicts RT sensitivity in EC.

mTOR is a downstream effector of the PI3K/AKT signaling pathway that regulates the production of nutrients for the cell [23]. mTOR dysregulation is implicated in the tumorigenesis of many cancers, however, this is unclear for EC. The activity of mTOR has not been associated with a more aggressive phenotype in EC [24,25]. Regarding RT, little is known about the effects of RT in mTOR activation and the role of mTOR in RT sensitization in EC except for Kourea’s study, which found a lack of correlation between the expression of phosphorylated mTOR in tissue microarrays of type 1 EC and RT use [25].

In order to determine the impact of mutations in the PTEN/PI3K/AKT pathway in EC, we performed an in silico analysis using public data sets from cBioPortal for cancer genomics including 1638 patients (Supplementary Material). Our analysis shows that mutations in *AKT1* and in *AKT2* are detrimental for OS, and that mutations in *PTEN*, *PIK3CA*, *PIK3R1* and *AKT3* were beneficial for OS and PFS (Figures S1 and S2). In the literature, the prognostic significance of mutations in PI3K-related genes for survival in EC was unclear. Different works have suggested that *PIK3CA* mutations have a favorable [26], unfavorable [27] or a neutral effect [27,28] on patients’ survival. In addition, one study showed that single mutations in *PIK3CA* and *PIK3R1* were not a significant predictors of OS or PFS, after accounting for the tumor stage and grade [29]. *PTEN* mutations are associated with favorable pathological, clinical and molecular features rather than with increased metastatic potential in EC [15]. Specifically, loss of PTEN expression has been identified as an independent prognostic marker for favorable survival in EC [30]. Finally, *AKT1* mutations have been restrictedly found in high grade, advanced stage EC [31], suggesting a detrimental effect in EC prognosis.

### 3.2. MAPK Signaling Pathway

The mitogen-activated protein kinase (MAPK) superfamily has been linked to the growth factor-mediated regulation of diverse cellular events such as proliferation, senescence, differentiation and apoptosis. Oncogenic alterations in Ras/MAPK pathway occur in the form of activating *KRAS* mutations that have been detected in 6–16% of cases of endometrial hyperplasia and 10–31% of cases of EC, being significantly higher in the MSI-positive ones [32]. Mutant *KRAS* promotes upregulation of MAPK and PI3K/AKT kinases, which further results in excessive cell proliferation and subsequently carcinogenesis. Several inhibitors of all these molecules, such as PD325901 or AZD6244, have been studied in EC and have been the subject of several anticancer targeted therapies [33,34].

Exposure of cells to ionizing radiation and a variety of other toxic stresses induces simultaneous compensatory activation of multiple MAPK pathway members controlling cell survival and repopulation effects following irradiation. Some of the signaling pathways activated are those normally activated by mitogens, such as the ‘classical’ MAPK (also known as the ERK) pathway, but also those downstream of death receptors, procaspases, and DNA-damage signals, including the JNK and P38 MAPK pathways [35]. Thus, the ability of radiation to activate MAPK signaling pathways may depend on the expression of multiple growth factor receptors, autocrine factors and *RAS* mutations, which basal expression can provide radiosensitivity or radioprotection. Specifically, in EC, two studies have related the MAPK pathway with ionizing radiation, both with the MEK inhibitor UO126. The first one was performed to predict radioresistance in EC cell lines, and the conclusion was that UO126 did not enhanced the effects of ionizing radiation, contrary to the PI3K/mTOR inhibitor NVP-BEZ235 [20]. The second work conducted for Marampon et al. was focused on the potential role of MEK/ERK inhibition on suppressing arising radioresistance mechanisms after irradiation [36]. There, treatment with U0126 increased radiosensitivity in a panel of gynecological cancer cell lines, with effects already evident at lowest radiation doses, suggesting that ERK inhibition enhances the response to DNA damage by radiation.

MYC is a transcription factor that can get activated by the MAPK pathway. MYC overexpression is highly prevalent in human malignancies [37,38]. In type 1 EC, *MYC* amplification has been associated with advanced and poorly differentiated disease [39]. Manning et al. have shown decreased *MYC* levels in the total blood in patients undergoing RT over the course of the fractionated schedule [40], the significance of which was not ascertained.

### 3.3. NF-κB Signaling Pathway

NF-κB is a family of dimeric transcription factors encoded by five genes: *NF-κB1*, *NF-κB2*, *RelA* (or *p65*), *c-Rel* and *RelB*. *NF-κB1* and *NF-κB2* produce the precursor proteins p105 and p100, respectively that when proteolyzed by the proteasome form the mature proteins p50 and p52, respectively. Usually, the different dimers combinations are kept inactive in the cytoplasm facilitated by the inhibitory proteins IκBs. With the proper stimulus, IκBs get phosphorylated and consequently undergo proteasomal degradation liberating the NFκB dimers, which are then able to translocate to the nucleus to activate NFκB-dependent transcription [41].

The NF-κB signaling pathway is constitutively activated in many human malignancies [42] including EC [43]. It is widely known that chemotherapy and radiation increase NF-κB signaling and that NF-κB plays a role in chemo- and radioresistance in human cancers favoring tumor unresponsiveness, progression and metastasis [44]. This is in agreement with the work performed by Santacana and coauthors, where they found significantly higher nuclear translocation and thus activation of p50, c-Rel and RelB in post-radiation vaginal recurrences (PVRs) compared to primary ECs by immunohistochemistry (IHC) [45]. However, short time frames of 24 h of ionizing radiation (1, 1.5 and 3 Gy) failed to significantly increase NF-κB transcriptional activity in Ishikawa cells [19,45]. In contrast, the blood of patients with EC subjected to RT present significant upregulation of *BCL2L1* mRNA levels 24 h after the 25th fraction of RT (5 weeks from the first fraction) compared to 24 h after the first or the second fraction [40]. *BCL2L1* encodes for Bcl-X_L_ and Bcl-X_S_, which are NF-κB targets that act as an antiapoptotic and apoptotic proteins, respectively [46]. This suggests that long RT exposure activates the NF-κB pathway.

Another factor that has been well established since the early 50s to trigger RT resistance is hypoxia, due to the inability to generate DNA peroxides, which are the result of the interaction of reactive oxygen species (ROS) generated during water radiolysis with oxygen and the DNA [47]. Tumor hypoxia occurs when there is insufficient oxygen supply to the growing tumor despite the erratic and abnormal neo-vascularization developed to meet the demands. Many strategies have been investigated preclinically to target hypoxia and reverse radioresistance by means of increasing the delivery of oxygen, radiosensitizing hypoxic cancer cells or selectively killing them. However, only one made it to the clinic, nimorazole, an oxygen mimetic, for the treatment of head and neck cancers [48]. Hypoxia is controlled by hypoxia-inducible factor 1α (HIF-1α). In EC, tumor vascularization and HIF-1α expression have been associated to higher mortality in patients [49,50]. Preclinical studies in EC pointed out that the inhibition of PI3K/mTOR and subsequent HIF-1α and VEGF-A downregulation increased the sensitivity to radiation in EC cell lines [20]. HIF-1 is activated by IKKβ [51]. Our group has previously discovered that hypoxia induces the translocation of p65 and p52 to the nucleus thereby activating both canonical and alternative NF-κB pathways in EC [52]. The processing and consequently activation of p52 occurs in the absence of HIF-1α, unlike p65 activation [52]. HIF-1α is highly expressed in recurrent ECs [53] including PVRs [45], where predominantly has a nuclear staining, compared to primary endometrial tumors. However, HIF-1α does not translocate into the nucleus after 60 min of 1 and 3 Gy of radiation in Ishikawa cells [45]. All these studies suggest that the NF-κB pathway and hypoxia participate in the long-term process of adaptation of tumors to RT and that hypoxia precedes such adaptation. Upon radiation treatment, hypoxia activates NF-κB, which leads to radioresistance prompting EC to reoccur.

### 3.4. Growth Factor Receptors and Growth Hormone

Growth factor receptors (GFR) are usually transmembrane proteins. They consist of an extracellular part responsible for a growth factor (GF) binding, a transmembrane part and an intracellular part that holds catalytic activity. The majority of GFR are receptor tyrosine kinases (RTKs). Others, like the TGF-β receptor are serine-threonine kinases. The binding of the GF to the receptor leads to its activation and represents the first step in the initiation of cell proliferation and differentiation cascades. Gain-of-function mutations, amplification and epigenetic changes in GFR have been implicated in cancer initiation and progression. It has been shown that GFR alterations confer resistance to RT in several cancers, such as epidermal GFR (EGFR) in glioblastoma [54] or fibroblast GFR (FGFR) in squamous cell carcinoma [55]. In EC, the role of GFR in inducing radioresistance is not well understood and it has been limitedly addressed.

We have recently demonstrated that ionizing radiation enhances the phosphorylation of the RTK c-Kit in Ishikawa cells and that the multi-RTK inhibitor Sunitinib abrogated such phosphorylation and rendered cells more sensitive to RT, suggesting a primordial role of c-Kit in sensitizing c-Kit+ ECs to RT [19]. c-Kit also seems important for the progression of EC as 25% and 40% of recurrent endometrioid carcinomas and uterine papillary serous carcinomas, respectively, presented positive staining for c-Kit while this RTK was absent in primary tumors [56].

EGFR is overexpressed in 46% and 34% of type 1 and type 2 ECs, respectively [57]. Some works suggest that EGFR overexpression could confer RT resistance and its inhibition could promote radiosensitivity in EC. For example, Shi et al. observed that EC xenografts made of radiation resistant cells slowed down their growth when EGFR was knocked-down by shRNA [58]. Additionally, photodynamic therapy with the photosensitizer radachlorin, able to generate ROS upon laser irradiation, downregulated the protein levels of EGFR in the EC cell line HEC-1-A 48 h after irradiation, concurrently with caspase-9 activation [59].

Regarding VEGFR, a study has shown higher expression of phosphorylated VEGFR2 in 44.5% of endometrioid carcinomas by IHC, which was linked with higher VEGF levels in patients’ total blood undergoing RT compared to benign disease and control patients [60]. Our group discovered expression of VEGFR2 in two out of the four EC cell lines analyzed (Ishikawa and KLE). Interestingly, these two cell lines were also resistant to ionizing radiation and Sunitinib induced RT sensitization [19]. In a similar manner to EGFR, photodynamic therapy reduced the expression of VEGFR2 at 8 h post-irradiation and effectively induced apoptosis in HEC-1-A cells [59]. The utility of VEGF inhibition as a radiosensitizer in EC was demonstrated in a clinical trial (NCT00545792) with 15 women with recurrent EC that were treated with the humanized monoclonal antibody bevacizumab with concurrent RT [61]. Notably, none of them experienced local relapse within the radiated field during a median of four-year follow-up [61].

**Figure 1:**
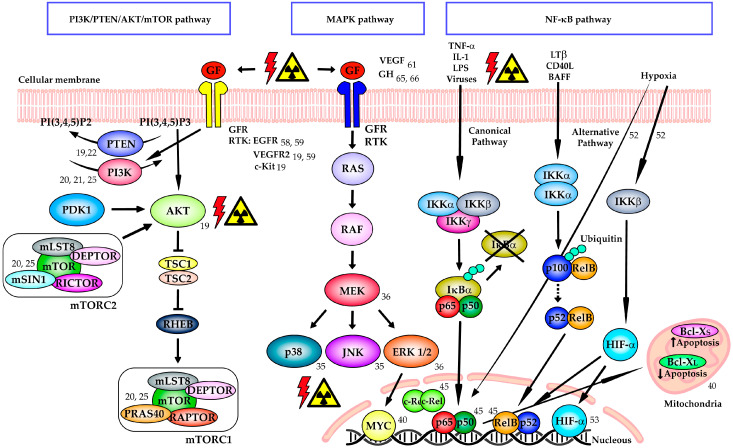
Participation of PI3K/PTEN/AKT/mTOR, MAPK and NF-κB signaling pathways in sensitivity to radiotherapy in endometrial cancer. Schematic representation of the signaling pathways and some of their members playing a role in sensitivity to ionizing radiation in endometrial cancer. References of the studies performed in endometrial tissues, cells or animal models are indicated for each target. Abbreviations: GF: growth factor, GFR: growth factor receptor, RTK: receptor tyrosine kinase, TNF-α: tumor necrosis factor alpha, IL-1: interleukin-1, LPS: lipopolysaccharide, LTβ: lymphotoxin-beta, CD40L: cluster of differentiation 40 ligand, BAFF: B-cell activating factor.

Growth hormone (GH) or somatotropin is a 191-amino acids peptide hormone secreted by the anterior pituitary gland and extra-pituitary tissue that stimulates growth and metabolism. High human (h)GH expression has been associated with lymph node metastasis, higher tumor stage, grade, myometrial invasion as well as worse prognosis in EC [62]. RL95-2 cells forcibly expressing hGH displayed a more aggressive phenotype and tumorigenic capacity in murine xenografts [63] as well as sensitivity to mitomycin C-induced DNA damage in vitro [64]. Bougen et al. demonstrated that treatment with the hGH receptor antagonist B2036 and ionizing radiation (4 Gy) synergistically reduced cell clonogenic survival and increased radiation-induced-DNA damage in RL95-2 cells [65]. One step further was done in 2016 by Evans et al. with the utilization of a pegylated form of B2036 (pegvisomant, Pfizer) in a RL95-2 xenograft model. The group demonstrated that the subcutaneous administration of pegvisomant at 100 mg/Kg significantly delayed tumor regrowth following exposure to fractionated ionizing radiation in mice through an unclear mechanism [66]. A representation of the above-mentioned signaling pathways playing a role in RT sensitivity can be found in Figure 1.

### 3.5. DNA Repair Mechanisms

**Figure 2:**
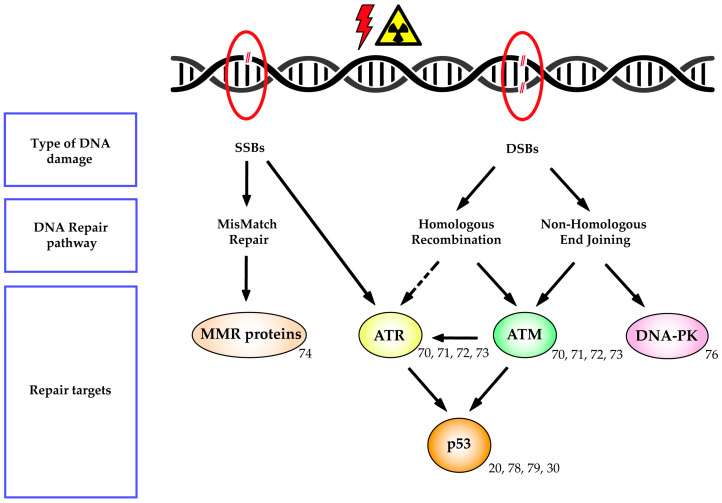
Activation of DNA repair mechanisms and their involvement in radiotherapy sensitivity in endometrial cancer. Scheme of the DNA repair pathways and targets activated by ionized radiation in EC. References of the studies performed in endometrial tissues or cells are indicated for each target. Abbreviations: single-strand breaks (SSBs), double-strand breaks (DSBs), mismatch repair (MMR), ataxia telangiectasia mutated (ATM), ataxia telangiectasia and Rad3-related (ATR), DNA-dependent protein kinase (DNA-PK).

RT induces cell death mostly due to DNA damage, especially DSBs. Consequently, tumor cells with highly efficient DNA damage response (DDR) machineries are radioresistant, whereas cancer cells with deficient DNA repair pathways are radiosensitive [67]. Therefore, therapies that inhibit the DNA repair process have the potential to enhance RT efficacy [68] (Figure 2).

Key regulators of the DDR are ataxia-telangiectasia-mutated (ATM) and ataxia telangiectasia and Rad3-related (ATR) proteins. ATM responds primarily to DSBs whereas ATR protects the integrity of replicating chromosomes. They belong to the class-IV phosphoinositide 3-kinase-related kinase (PIKK) family and their activation induces phosphorylation of its downstream targets, such as Chk1 (checkpoint kinase 1) or Chk2 (checkpoint kinase 2), and regulates cell cycle and DNA repair [69]. Various studies in EC cell lines evidenced that pharmacologic inhibition of ATR and ATM enhances the cytotoxic effect of radiation [70,71]. Moreover, homologous recombination (HR), mainly promoted by ATM and ATR, deficiency is common in ECs as proved from the results obtained using a functional RAD51-IRIF assay, where several EC samples were irradiated to induce DNA DSBs [72]. HR is also associated with cervical and endometrial tumor response to RT [73].

The MMR system is responsible for repairing base mismatches and includes the MMR proteins hMLH1, hMSH2, hPMS2, hMSH3 and hMSH6. Aberrations in MMR genes are involved in carcinogenesis of EC type I and mutations in *hMLH1* have been detected in 20–40% of EC cases [32]. Regarding RT, a recent paper concluded that adjuvant RT improved survival in EC MMR-deficient patients and that MMR status could be used as a predictive biomarker to select patients that benefit most from adjuvant RT [74].

DSBs, apart from HR, could be repaired by non-homologous end joining (NHEJ) activated by the DNA-dependent protein kinase (DNA-PK) complex, among other factors. DNA-PK is a trimetric enzyme consisting of a 460-kDa catalytic subunit (DNA-PKcs) and a heterodimeric regulatory complex called Ku, which is composed of 70 kDa protein (Ku70) and 86 kDa protein (Ku80) subunits [75]. Low Ku70 expression is associated with better PFS in EC, probably due to a major sensitivity of tumors to RT [76].

p53 is a tumor suppressor known to be post-translationally modified, stabilized and activated in response to cellular stress such as DNA damage, oncogene expression or ribosome dysfunctions and activates the transcription of numerous genes implicated in cell cycle arrest, DNA repair, apoptosis and senescence [77]. The *TP53* gene is mutated approximately in half of human cancers and in 90% of type II ECs, but only in 10–20% of Grade 3 type I ECs [32]. The effects of cellular exposure to irradiation on p53 activation are well-known but studies deciphering the role of p53 status in RT sensitivity in EC have given contrasting results. An old study did not correlate *TP53* mutations with radiosensitivity in gynecological cancer cell lines [78]. Similarly, women with endometrioid carcinomas with *TP53* alterations did not show differences in OS after receiving adjuvant RT compared to women with wild-type TP53 [79]. In contrast, other experiments in EC cell lines demonstrated that wild-type *TP53* cell lines were more sensitive to radiation than the *TP53*-mutated ones [20]. In the same line, another work demonstrated significant p53 overexpression in type 2 EC, which was associated with advanced stage and poorer OS after adjuvant RT [30]. Conversely, one study confirmed that patients with endometrioid carcinomas harboring *TP53* alterations benefited greater from adjuvant RT than the wild-type ones [79].

### 3.6. Immune System

The first studies establishing associations between the immune system and RT in EC indicated that RT decreased peripheral blood T-cell numbers [80]. Nevertheless, now is recognized that a component of tumor damage after radiation may be due to immunogenic cell death. Radiation induces immunostimulation through the release of tumor antigens, cytokines, chemokines and increases expression of death receptors and other molecules that contribute to recognition of tumor cells by the immune system [81]; and most importantly, alters the microenvironment within the irradiated field, increasing the density of tumor infiltrating lymphocytes (TILs).

Therapies based on immune checkpoint inhibitors, mainly the cytotoxic T-lymphocyte-associated protein 4 (CTLA-4) and programmed-death receptor 1 (PD-1), demonstrated significantly poorer response rates in gynecologic tumors [82,83] with the remarkable exception of PD-1 inhibitors for the treatment of microsatellite instability-high (MSI-High) or mismatch repair-deficient (dMMR) metastatic ECs [84]. These two subgroups have been shown to possess high neoantigen loads and increased number of TILs together with PD-1 and programmed death-ligand 1 (PD-L1) overexpression [85]. For this reason, the combination of immunotherapy with radiation in EC has recently generated great interest. The immunologic properties of radiation may complement the immune stimulatory effects of both CTLA-4 and PD-1 pathway inhibition, as preclinical studies have demonstrated synergies with both classes of agents and radiation in terms of improved local and distant control, including abscopal regression of established tumors outside of the radiation treatment field [86]. Based on these findings, a wide range of RT doses and fractionation schedules have been proposed for optimal combination with immunotherapy in studies deploying mice models. There, it is shown that the efficacy of low doses of fractionated RT can be enhanced when delivered in combination with antibodies against PD-1 and PD-L1 [87]. Currently, there are several ongoing clinical trials in patients with advanced uterine cancers testing monoclonal antibodies against key immune checkpoint inhibitors, such as durvalumab (PD-1), pembrolizumab (PD-1), tremelimumab (CTLA-4) or TSR-042 (PD-1), administrated simultaneously or sequentially to RT [88].

### 3.7. Others

Besides the signaling pathways and proteins exposed above, there are other unrelated markers that have a role in RT sensitivity that deserve special attention. One of these is *PTTG*, the pituitary tumor transforming gene. *PTTG* is involved in many biological and tumoral processes including chromatid separation, DNA repair, organ development, angiogenesis and metastasis. PTTG has been found overexpressed in brain tumors and pituitary adenomas [89,90]. Silencing *PTTG* expression by siRNA inhibited the growth of the EC cell line HEC-1A and potentiated the effects of RT in cell growth inhibition and apoptosis induction [91]. Another important biomarker is Hsp70, product of the *HSP70* gene, a heat shock protein involved in protein folding and expressed under cellular stress. Hsp70 has often been found to be overexpressed in cancer cells because they rely on its protective survival properties [92]. Silencing *HSP70* expression enhances the therapeutic effect of RT in EC cells [93]. The G2/M checkpoint is another interesting target in cancer as many tumors are defective in G1/S checkpoint and rely on G2/M for cell division and survival. It is fairly screened for drugs, particularly those targeting Plk and Chk proteins. Plk1 is found overexpressed in EC tissue [94] and abrogation of *Chk1*, *Chk2* and *Plk1* expression in EC cells potentiates the antitumor efficacy and apoptosis induced by cisplatin or radiation [94,95]. Another interesting protein related to RT response is osteopontin-1 (OPN). OPN, encoded by the *SPP1* gene, is a secreted phosphorylated glycoprotein initially found in the extracellular matrix of the bone and widely expressed across tissues [96]. OPN has also been identified as a hypoxia-responsive protein, being associated with radiation resistance mechanisms in lung and breast cancer cells [97,98]. In EC, inhibition of OPN renders EC cells more susceptible to RT [99]. In the clinical setting, elevated OPN levels in plasma predict poor disease outcomes in patients with head and neck cancer [100] and breast cancer [101].

EC is a hormone dependent disease where the expression of estrogen receptor (ER) and progesterone receptor (PgR) has been associated with histological tumor differentiation, response to therapy and metastatic potential. In particular, loss of ER, PgR-A and PgR-B are key events of endometrial carcinogenesis and the predominance of some isoforms respect others can explain some clinical features [102]. For example, patients with a ratio PgR-A/Pgr-B lower than 1 have shorter OS and PFS [103]. In general, patients with ER+ and/or PgR+ tumors present a good response to hormone therapy and could even confer radiosensitivity. Primary explants of highly differentiated ECs showed enhanced radiosensitivity when treated with medroxyprogesterone, a progestogen [104].

The *ARG1* gene encodes for arginase 1 enzyme, which controls the last step of the urea cycle, where arginine is hydrolyzed to form ornithine and urea. Recent studies confirm that *ARG1* is expressed in activated M2 macrophages and participates in anti-inflammation, tumor immunity, tumor proliferation, metastasis and immunosuppression-related diseases [105]. One study found upregulation of *ARG1* expression in EC patients’ total blood, which was sustained at least 5 weeks after the first irradiation delivery, suggesting that *ARG1* expression level could be a predictive biomarker of late RT irradiation exposure [40].

It has been described that efflux pumps play a role in chemotherapy and RT resistance. One example is P-glycoprotein, a membrane protein ATP-driven drug efflux pump, which is associated with multidrug resistance and found overexpressed in many tumors [106] including EC [107]. P-glycoprotein confers resistance to RT and suppresses radiation-induced apoptosis in ovarian and lymphoma cancer cells [108]. Cancer stem cells (CSCs) also overexpress drug efflux pumps and have an enhanced DNA damage repair machinery and activation of mitogenic and antiapoptotic pathways, altogether leading to chemotherapy and RT resistance [109]. It has been postulated that the remaining CSC present after RT administration could be responsible of cancer relapse [110]. In EC, CSCs play a role in EC initiation, metastasis and overall chemo- and radioresistance through the activation of the epithelial-to-mesenchymal transition (EMT) program, Hedgehog, PI3K/AKT/mTOR and NOTCH signaling pathways [111]. However, possible radiosensitization effects resulting from the suppression of CSC have not yet directly been evaluated in EC. In this line, we demonstrated that inhibition of c-Kit signaling, a CSC marker [111], by Sunitinib sensitizes Ishikawa cells to ionizing radiation [19]. Additionally, PTEN/PI3K/AKT/mTOR pathway favors stemness through the upregulation of EMT inducers [112] and its inhibition induces radiosensitization [20].

## 4. Functional Assays that Assess Radiosensitivity

The most commonly used in vitro functional assays for assessing radiosensitivity are clonogenic assays, determination of micronuclei frequencies and detection of γ-H2AX. Clonogenic assays determine the ability of cancer cells to form colonies (>50 cells) over a certain period of time, usually 1–3 weeks [113]. After a radiotherapeutic insult, such an ability is compromised [19]. The clonogenic assay provides the fraction of reproductive cells remaining after irradiation. The sensitivity of the assay is high as it detects minor changes in survival; the effect is expressed in the logarithmic scale. The specificity is low, as other treatments, such as chemotherapeutic drugs, can reduce clonogenicity. One limitation of the clonogenic assays is the long time required for the obtainment of the result. Additionally, the assay is unable to differentiate between dead and non-proliferating colonies. The formation of radiation-induced micronuclei refers to the formation of chromatin particles originated from whole chromosomes or fragments of them during mitosis as a result of radiation [114]. Similar to clonogenic assays, tests aiming to determine micronuclei have high sensitivity and low specificity. Micronuclei can easily be detected by in situ hybridization (FISH) of centromeres or by regular nucleic acid stains [115]. However, one limitation is its low specificity since the formation of micronuclei occurs both under normal physiologic conditions and drug treatments. γ-H2AX is a known marker of DNA double-strand breaks, which are one of the consequences of ionizing radiation. This phosphorylation in histone H2AX can be detected and quantified by several techniques including Western blot or immunofluorescence [19]. γ-H2AX is a measure of radiosensitivity and disappears once the cell repairs the DNA damage. The γ-H2AX analysis possesses high sensitivity as the detection involves the use of antibodies conjugated usually to fluorophores [19]. Additionally, it has higher specificity compared to clonogenic assays and micronuclei tests due to the detection of a very specific type of DNA damage. However, the transiency of the phosphorylation in H2AX is a limitation of the technique. In addition, the assay is unable to distinguish between faithful DNA repair and misrepair and between simple and complex DNA damage [116].

## 5. Current Clinical Trials Employing Agents with Radiosensitization Properties in Endometrial Cancer

The choice of adjuvant treatment for each EC’s risk group is supported by the results of clinical trials. However, it is still today a matter of intense debate [117]. Multiple untargeted drugs have been studied in combination with RT for EC treatment. These include taxanes (docetaxel and paclitaxel), platinum compounds (cisplatin and carboplatin), anthracyclines (doxorubicin), nucleotide analogs and precursors (gemcitabine, capecitabine and 5-fluorouracil) and vinca alkaloids and derivatives (vinorelbine) [118,119,120]. In contrast, a lack of targeted adjuvant treatments is emphasized.

In this section, we will discuss the targeted therapies utilized for inhibiting or activating cellular pathways known to play a role in radiation resistance or sensitivity, in combination with RT in EC. There are currently ten clinical trials, which have evaluated or are evaluating the efficacy of such targeted therapies (Table 1). These can be classified in GF/GH inhibitors (bevacizumab and octreotide), PARP inhibitors (talazoparib) and immune system modulators (filgrastim, pegfilgrastim, pembrolizumab, durvalumab, tremelimumab and TSR-042). We will also discuss in this section the most recent clinical trials, either recently completed or currently active, employing chemotherapy in combination with radiotherapy in EC (Table 2).

Some studies have studied tolerability, efficacy and secondary effects of GF with RT. A phase II trial (NCT00545792) has assessed the anti-VEGF antibody bevacizumab with or without RT in recurrent pelvic-confined gynecological cancers, including EC. Results from this trial concluded that the delivery of bevacizumab with concurrent radiation provides excellent local tumor control and survival, particularly in those patients with unresectable nodes [61]. Later, another phase II trial (NCT01005329) investigated the administration of intensity-modulated radiation therapy (IMRT) in combination with bevacizumab and cisplatin, followed by carboplatin and cisplatin, for efficacy and secondary effects in patients who had undergone surgery for high-risk EC. Results from this clinical trial revealed that postoperative bevacizumab added to chemotherapy and pelvic IMRT appeared to be well tolerated and resulted in high OS rates after two years in patients with high-risk EC [61]. Finally, an older phase III clinical trial (NCT00033605) published in 2008 investigated the utility of octreotide, a somatostatin analog able to inhibit GH, in reducing diarrhea in patients undergoing pelvic radiation as treatment of various cancers, including EC [121]. The authors concluded that octreotide not only did not decrease diarrhea but also worsened gastrointestinal symptoms in some patients. Thus, the administration of octreotide was not indicated, despite previous shown success in reducing severe liquid bowel content [122]. The clinical trial did not assess radiosensitization effects due to octreotide.

Regarding PARP inhibitors, an ongoing phase I trial (NCT03968406) is determining optimal drug dose, safety and tolerability of talazoparib in combination with RT in patients with gynecologic cancers, including EC that have relapsed after previous treatment. The trial aims to analyze incidence of side-effects, response rate, local control rate, time to progression, PFS, OS, level of PARP inhibition, ɣ-H2AX and RAD51 foci formation levels and overall quality of life, among other variables.

Immunotherapy constitutes a new paradigm of novel therapies with promising radiosensitizing properties. In 2003, a phase III trial (NCT00006011) started to analyze the effect of the immune system activators filgrastim and its pegylated version, pegfilgrastim, in combination with cisplatin, doxorubicin and ± paclitaxel, in previously irradiated EC patients. Filgrastim induces the proliferation of neutrophil progenitor cells and it is used for the treatment of chemotherapy-induced neutropenia [123]. The investigators of the trial concluded that the addition of paclitaxel to cisplatin and doxorubicin following surgery and radiation was not associated with a significant improvement in recurrence-free survival but with increased toxicity [124]. More recently, novel proposed immunotherapies in combination with RT have just started to emerge for EC treatment. Currently, there are five clinical trials in recruitment status that aim to assess the efficacy, the maximum tolerated dose of the drug and side-effects of RT with immune checkpoint inhibitors, in particular monoclonal antibodies against PD-1, PD-L1 or CTLA-4. The first one is a phase II trial with pembrolizumab (anti-PD-1), vitamin D, aspirin, cyclophosphamide, lansoprazole and curcumin administered concurrently with EBRT in advanced or refractory EC (PRIMMO, NCT03192059). This study aims to primarily determine overall response rate at week 26. The second one is a phase I clinical trial using pembrolizumab administered before chemoradiation treatment (paclitaxel and carboplatin and, BT) in high and intermediate-risk EC patients (FIERCE, NCT03932409). This study intends to primarily determine the proportion of patients completing three cycles of pembrolizumab combined with dose dense paclitaxel and carboplatin chemotherapy during a time frame of 36 months. The third one is a phase III clinical trial testing pembrolizumab with EBRT and BT in stage I-II EC patients that are mismatch repair deficient. Pembrolizumab is administered seven days prior to RT. The primary outcome of the trial is to compare the three-year RFS of patients treated with RT plus pembrolizumab versus RT alone. The fourth one is a phase I clinical trial deploying durvalumab (anti-PD-L1) and tremelimumab (anti-CTLA-4) with concurrent EBRT in metastatic or unresectable ECs (NCT03277482). The primary outcome of this study consists of determining the maximum tolerated dose of RT with durvalumab and tremelimumab during a time frame of eight weeks as well as the incidence of dose-limiting toxicities for each dose level or regimen. Finally, the fifth one is a phase I clinical trial employing TSR-042 (anti-PD-1) administered before BT in inoperable stage I/II EC patients (NCT03955978). The primary outcome of the trial is to assess safety and tolerability of TSR-042 with BT measured by the grade of toxicities experienced by the patients during a time frame of six weeks. We expect the results of these four clinical trials to be reported in the near future.

Apart from the targeted therapies used in combination with RT, we have identified seven recently closed or currently active clinical trials employing chemotherapy plus RT in EC that are worth mentioning. The first is a phase II study (NCT00285415) completed in September 2019, which aimed to determine the effectiveness of the combination of carboplatin and docetaxel followed by tumor volume directed pelvic plus or minus para-aortic irradiation in stage III/IV or recurrent EC. Its primary objective was the estimation of the overall response rate of the therapy. The second is a phase II clinical trial (NCT03935256), which is evaluating the safety of sequential and concurrent carboplatin and paclitaxel with adjuvant EBRT for locally advanced EC. Its primary objective is to assess acute grade 3-4 non-hematologic and grade 4 hematologic toxicities associated with the above regimen. The third is a phase II study (NCT04386993), namely DeCRESCEndo, which will assess the benefit of short course radiation in post-operative stage III-IVA EC patients. The investigators hypothesize that short course pelvic radiation will benefit patients from both convenient and effective loco-regional control comparable to the traditional 5-6 weeks of radiation, and will have low acute and late grade 3–4 toxicity rate (<10%). The fourth is the phase II trial (NCT00411138) including high-risk stage I-III EC patients, which intends to study the efficacy on the administration of chemotherapy and RT compared to RT alone. The fifth is a phase III trial (NCT00942357) that aimed to assess the efficacy of carboplatin and paclitaxel in with or without cisplatin and RT in stage I-IVA EC patients. Results from the trial revealed that chemotherapy plus RT was not associated with longer relapse-free survival compared to chemotherapy alone [13]. The sixth is a phase III trial (NCT00807768) including patients with high-risk stage I or stage II EC that has assessed the efficacy of pelvic RT compared to vaginal implant RT, paclitaxel and carboplatin. Results failed to demonstrate the superiority of vaginal RT compared with pelvic RT. In addition, although late toxicity was similar in both arms, acute toxicity was greater in the vaginal RT-treated one. Authors concluded that pelvic RT alone remains an effective, well-tolerated and appropriate adjuvant treatment in high-risk early-stage EC for all histologies [12]. Finally, the seventh study is a phase II trial (NCT00492778) that is evaluating the efficacy of RT and cisplatin compared to RT alone in patients with recurrent EC.

Taking into account all these observations, we think that there are two promising strategies in the horizon likely to be implemented in the clinic. On the one hand, there is a clear interest in investigating immune checkpoint inhibitors in combination with RT in EC; five out of the six active clinical trials employing targeted therapies are utilizing immunotherapies (Table 1). Although these clinical trials have not yet reported results, there is evidence that the strategy has worked in improving OS and PFS in metastatic disease of several cancers including EC [125]. On the other hand, we identified that the foremost recent studies with chemo- and RT in EC investigate modern variants of radiation therapy such as proton therapy, intensity-modulated radiation therapy (IMRT) and volume-modulated arc radiotherapy (VMAT), alone or in combination with platinum compounds (Table 2), with results yet to be published in most of the cases. Such modern therapies have the advantage of reducing off-target irradiations [126] and associated secondary effects in EC [127,128]. Moreover, IMRT and VMAT, alone or combined with chemotherapy, have successfully reduced the risks of recurrence and death in non-endometrioid EC. Patients over 60 years of age as well as those with endometrioid histology, lymphovascular space invasion, and with two or more positive lymph nodes benefitted the most [128].

## 6. Cutting-Edge Advances in Radiotherapy for Endometrial Cancer

The latest advances in RT and its closely related fields for EC encompasses several experimental approaches that include modern RT techniques and radiogenomics.

### 6.1. Modern Radiotherapy

RT techniques used for EC treatment are divided into external or internal BT depending on the localization of the RT source. Modern external RT techniques that have emerged in the past few decades include IMRT, VMAT and stereotactic body radiation therapy. IMRT uses multiple x-rays beam of different intensities and angled from different directions with the objective to adapt better to the irregular shape of tumors. In EC, this type of RT prevents organs of the pelvic region such as the bladder and rectum to be excessively irradiated, decreasing related morbidities and side effects. This method is always used with image-guided RT (IGRT), which consists of the imaging of the tumor by computed tomography (CT) scan or x-rays and allows for a very precise targeting of the tumor in three dimensions (3D). One step forward is the addition of 4D in IGRT, which enables the adjustment of the beams in real-time during the treatment. Disadvantages of IMRT are the increased risk of secondary malignancies induced by the radiation and the longer time needed for delivering the radiation, which VMAT have tried to overcome [129]. VMAT, which was introduced in 2007, consists of the delivery of the radiation by a source that continuously rotates 360°. Both IMRT and VMAT have a good balance benefit/risk in EC compared to conventional conformal RT [129]. Like EBRT, BT has evolved into intensity-modulated BT (IMBT), 3D and 4D-IGBT, having the same promises and drawbacks as IMRT and IGRT [130]. Both 3D and 4D-IGBT have shown good local control for women with recurrent EC [131,132] and a 3D-EGBT high response rate in inoperable early stage endometrioid EC [133]. Finally, a novel RT emerging for the treatment of EC is proton therapy. Proton therapy uses protons that are accelerated by a synchrotron. The advantages are that it allows higher doses of radiation in the tumor while it reduces irradiation of nearby healthy tissues up to 60%, which decreases secondary effects. Its use, however, is not as widely available in the hospitals as conventional RT. A clinical trial currently in recruiting status will assess toxicity as well as PFS and quality of life of proton therapy in 25 patients with uterine cervical and endometrial malignant neoplasms (NCT03184350). Patients will receive a fractionated dose of 45–50.4 Gy 5–6 times per week using an active raster-scanning pencil beam proton radiation.

### 6.2. Radiogenomics

Alongside the progress of genomic technology, radiogenomics has emerged as a novel discipline for the discovery of biomarkers for the prediction of RT response. The identification of such biomarkers is still in its infancy in EC. Currently, informed decisions about RT administration in EC are mainly based on clinical and histopathological characteristics that are unable to fully explain the RT responses. However, some works have brought light into the genes and mechanisms that determine EC sensitivity to RT. Yard et al. profiled 533 genetically annotated human cancer cell lines and identified genetic determinants of the response to radiation [21]. They found an association between radiation sensitivity and low copy number alterations, high frequency of mutations and mutations in DNA repair genes in EC cell lines [21]. Interestingly, most of the cell lines harboring mutations in the p85-binding domain of PIK3CA were sensitive to radiation and 38% belonged to the uterine lineage. Additionally, a recent genomic stratification has helped the treatment decision in EC [5]. This study has revealed frequent incorrect IHC subtyping between high-grade type 1 and type 2 ECs; and also, inappropriate allocation of RT in endometrioid copy-number high ECs when they would better benefit from chemotherapy.

## 7. Conclusions

EC presents generally very good prognosis except in the 20% of cases, which metastasize or locally relapse. RT is the first-line treatment after surgery for EC in early stages and plays a crucial role reducing the risk of recurrences. The clinical importance of RT in EC is witnessed in the literature with numerous entries. However, despite being so widely used in this cancer, the basic knowledge about the cellular processes and individual biomarkers that determine sensitivity or resistance to RT is rather limited. Currently, there is some general consensus about the positive association between PI3K/AKT/mTOR, MAPK, NF-κB, growth factor receptor and growth hormone activation and radioresistance; and between DNA repair machinery deficiencies and radiosensitivity in EC. Regarding the immune system, abrogation of both CTLA-4 and PD-1 pathways has been associated with greater radiosensitivity. Despite this existing knowledge, there is still a long way to go to fully understand the molecular determinants responsible for radiosensitivity in EC and whole-genome studies can be the perfect tool to unravel these unknowns. They have already provided extensive knowledge about the molecular characteristics in EC and a relatively recent subtyping scheme. In the future more sophisticated radiogenomics studies employing latest technologies, such as single-cell genomics, will allow the detection of dynamic changes during and after irradiation with associations between a positive RT response to gene and protein expression changes, which can be later targeted by highly selective therapeutic approaches. This will lay the foundation for personalized radiotherapy, which will improve treatment outcomes in EC.

## Figures and Tables

**Table 1:** Clinical Trials with Targeted Therapies with Radiosensitization Properties.

Trial Name, Identifier And [Status]	Phase	N	Official Name	Type of EC Included	Drugs and Treatment Scheme	Radiation Regimen and Schedule	Primary Outcomes	Secondary Outcomes
**Growth factors—Growth factor inhibitors**								
NCT01005329 [Completed]	II	34	A Phase II Study of Postoperative Intensity Modulated Radiation Therapy (IMRT) With Concurrent Cisplatin and Bevacizumab Followed by Carboplatin and Paclitaxel for Patients With Endometrial Cancer	Resected high-risk stage I-IV EC	Bevacizumab (anti-VEGF), and cisplatin, followed by carboplatin and paclitaxel	Pelvic IMRT once daily, 5 days a week, for 5 weeks (45 Gy in 25 fractions) with optional nodal boost RT and/or vaginal BT boost. Concurrently with bevacizumab+cisplatin.	Adverse events by grade within 90 days after the treatment starts	Adverse events by grade within 90 days within 1 year treatment start and from start of treatment to end of follow-up, up to 43.4 months; OS, DFS, pelvic failure rate and distant failure
NCT00545792 [Completed]	II	21	A Pilot Study Evaluating The Safety Of Avastin And Pelvic Radiation In Women With Pelvic-Confined Recurrence of Gynecological Cancers	Recurrent pelvic-confined EC	Bevacizumab	Daily Pelvic RT. Concurrently with bevacizumab.	PFS OS	Thrombosis and one embolic event in the setting of metastatic disease
NCT00033605 [Completed]	III	130	Phase III Double-Blind Study Of Depot Octreotide Versus Placebo In The Prevention Of Acute Diarrhea In Patients Receiving Pelvic Radiation Therapy	EC	Octreotide (GH inhibitor) Arm I: Short-acting octreotide SC on day 1 and long-acting octreotide IM on days 2 and 29. Arm II: Patients receive placebo SC on day 1 and IM on days 2 and 29	Prior planned cumulative dose of RT, including boost fields (4500–5350 cGy vs. 5351–6000 cGy vs. more than 6000 cGy) and planned intracavitary BT. RT starts maximum 4 days before octreotide.	Reduction of diarrhea measured weekly during pelvic RT up to 2 years	Reduction of patient-reported bowel dysfunction, toxicity and importance that patients attach to various measures of bowel dysfunction as assessed by questionnaire
**DNA repair pathway—PARP inhibitors**								
NCT03968406 [Recruiting]	I	24	Phase I Study of Talazoparib in Combination With Radiation Therapy for Locally Recurrent Gynecologic Cancers	Stage IV or recurrent EC	Talazoparib (PARP inhibitor)	Fractionated RT 5 days per week for up to 7 weeks. Concurrently with talazoparib.	MTD	Adverse events, RR, PFS, OS. Others: PAR inhibition levels, ɣ-H2AX and RAD51 foci formation levels, OQL
**Immunotherapy—Immune system activators and immune checkpoints inhibitors**								
GOG-0184 NCT00006011 [Completed]	III	659	A Randomized Phase III Study of Tumor Volume Directed Pelvic Plus or Minus Para-Aortic Irradiation Followed by Cisplatin and Doxorubicin or Cisplatin, Doxorubicin and Paclitaxel for Advanced Endometrial Carcinoma	Stage III or IV EC	Filgrastim (G-CSF analog) or pegfilgrastim (pG-CSF analog) Arm I: doxorubicin and cisplatin, followed by G-GSF or pGCSF Arm II: doxorubicin and cisplatin, followed by paclitaxel and followed by G-GSF or pGCSF	Pelvic or extended field RT. Within 8 weeks after surgery, patients receive tumor VDPR with or without PNR once daily for 5 consecutive days for up to 16 weeks after surgery. Within 8 weeks of completing RT, patients receive Arm I or Arm II.	RFS	Not provided
PRIMMO NCT03192059 [Recruiting]	II	43	A Phase II Investigation of Pembrolizumab (Keytruda) in Combination With Radiation and an Immune Modulatory Cocktail in Patients With Cervical and Uterine Cancer (PRIMMO Trial)	Advanced and refractory EC	Pembrolizumab (anti-PD-1) vitamin D, aspirin, cyclophosphamide, lansoprazole and curcumin	EBR 24 Gy in 3 fractions, a fraction every 8 h Concurrently with drug scheme.	ORR at week 26	Incidence of adverse events, ORR, best OR, PFS, OS and OQL assessment
FIERCE NCT03932409 [Recruiting]	I	20	A Phase Ib Trial of Vaginal Cuff Brachytherapy + Pembrolizumab (MK3475) Followed by 3 Cycles of Dose Dense Paclitaxel/q 21 Day Carboplatin + Pembrolizumab (MK3475) in High Intermediate Risk Endometrial Cancer	High and intermediate-risk EC	Pembrolizumab (anti-PD-1) and chemoradiation consisting of dose dense paclitaxel and carboplatin and, BT	Vaginal cuff BT given one week after pembrolizumab.	Patients completing 3 cycles of pembrolizumab	PFS OS Adverse event frequency
NCT04214067 [Recruiting]	III	168	A Phase III Randomized Trial of Radiation ± MK-3475 (Pembrolizumab) for Newly Diagnosed Early Stage High Intermediate Risk Mismatch Repair Deficient (dMMR) Endometrioid Endometrial Cancer	High-intermediate risk stage I-II dMMR EC	Pembrolizumab (anti-PD-1) Arm 1: EBRT + BT Arm 2: ERBT + BT + Pembrolizumab administered 7 days prior to the start of RT, every 3 weeks for up to 1 year (17 cycles)	ERBT daily for 5–6 weeks and vaginal BT completed within 7 days after completion of EBRT. Pembrolizumab given 7 days prior to the start of RT.	3-year RFS	Incidence of adverse effects, recurrence patterns, 5-year RFS, OS, patients reported outcomes
NCT03277482 [Recruiting]	I	23	A Phase 1 Study of Durvalumab, Tremelimumab and Radiotherapy in Recurrent Gynecologic Cancer	Recurrent and metastatic EC	Durvalumab (anti-/PD-L1) + Tremelimumab (anti-CTLA-4)	EBT hypofractionated short course (either 1 or 5 days). Concurrently with the immunotherapies.	MTD	ORR, LRR, LCR, ARR, RD, PFS, OS
NCT03955978 [Recruiting]	I	12	A Phase I Study of PD-1 Inhibition With TSR-042 in Addition to Standard of Care Definitive Radiation for Inoperable Endometrial Cancer	Inoperable EC	TSR-042 (anti-PD-1)	BT 36 Gy in 6 fractions, given weekly. The first dose of TSR-042 is administered 21 days prior to the first BT fraction.	Adverse event at six weeks	PFS

Abbreviations: ARR: abscopal response rate; BT: brachytherapy; CTLA-4: cytotoxic T-lymphocyte-associated protein 4; DFS: disease free survival; dMMR: deficient mismatch repair; EBT: external beam therapy; G-CSF: granulocyte-colony stimulating factor; IM: intramuscularly; Intensity-modulated radiation therapy: IMRT; IV: intravenously; LCR: local control rate; LRR: loco-regional recurrence; MTD: maximum tolerated dose; OQL: overall quality of life; ORR: overall response rate; OS: overall survival; PD-1: programmed cell death-1; PD-L1: programmed death-ligand 1; PFS: progression-free survival; PNR: para-aortic nodal radiotherapy; RD: response duration; RFS: recurrence-free survival; RR: response rate; RT: radiotherapy; VDPR: volume-directed pelvic radiotherapy; VEGF: vascular endothelial growth factor.

**Table 2:** Recently Closed and Active Clinical Trials Employing Chemotherapy Combined with Radiotherapy.

Trial Name, Identifier and [Status]	Phase	N	Official Name	Type of EC Included	Drugs and Treatment Scheme	Radiation Regimen and Schedule	Primary Outcomes	Secondary Outcomes
**Recently completed clinical trials**								
NCT00285415 [Completed]	II	46	A Phase II Evaluation of Docetaxel and Carboplatin Followed by Tumor Volume Directed Pelvic Plus or Minus Para-Aortic Irradiation for Stage III/IV Endometrial Carcinoma	Advanced stage III and pelvis-confined stage IV or recurrent EC	Docetaxel + carboplatin: every 3 weeks for 6 cycles	Tumor Volume Directed Pelvic ± Para-Aortic Irradiation. After chemotherapy	ORR	OS, PFS, safety and tolerability
**Active clinical trials**								
NCT03935256 [Recruiting]	II	24	Phase II Study of Concurrent and Sequential Carboplatin and Paclitaxel With Adjuvant Radiotherapy for High Risk Endometrial Cancer	Locally advanced stage III-IVA EC	Carboplatin + paclitaxel: Arm 1: carboplatin + paclitaxel 4 cycles weeks 1, 10, 13 and 16. Arm 2: carboplatin + paclitaxel 2 cycles weeks 4 and 7 + RT	EBPR of 45 Gy in 25 fractions followed by vaginal BT boost at doses of 12–18 Gy in 2–3 fractions. Sequentially and concurrent with carboplatin + paclitaxel	Acute toxicities	Treatment delays, chronic toxicities, local control, pelvic failure-free survival, distant metastasis-free survival, cause-specific survival, DFS and OS
DeCRESCEndo NCT04386993 [Not yet recruiting]	II	25	De-escalated Conformal Radiation Expedited Sequentially With Chemotherapy for Endometrial Cancer (DeCRESCEndo)	Stage III-IVA EC	Chemotherapy: regimen not determined	IMRT: 5-Gy in 5 fractions with elective simultaneous boost to any suspicious lymph node or residual disease to 30 Gy in 1–2 weeks. Sequentially with chemotherapy	Adverse effects incidence	Change in patient-reported toxicity, change in QOL, Loco-regional control, distant control, DFS and OS
PORTEC-3 NCT00411138 [Active, not recruiting]	III	670	Randomized Phase III Trial Comparing Concurrent Chemoradiation and Adjuvant Chemotherapy With Pelvic Radiation Alone in High Risk and Advanced Stage Endometrial Carcinoma: PORTEC-3	High risk stage I-III EC	Cisplatin + paclitaxel + carboplatin: Arm 1: EBPR combined with 2 cycles of cisplatin followed by 4 cycles of carboplatin + paclitaxel Am 2: ERBT and vaginal BT in case of cervical involvement	EBPR: 48.6 Gy in 1.8 Gy fractions up to 6 weeks and vaginal BT boost in case of cervical involvement. Alone or concurrently with chemotherapy	OS, failure-free survival	QOL, severe treatment-related morbidity, vaginal or pelvic relapse and distant metastasis
NCT00942357 [Active, not recruiting]	III	813	A Randomized Phase III Trial of Cisplatin and Tumor Volume Directed Irradiation Followed by Carboplatin and Paclitaxel vs. Carboplatin and Paclitaxel for Optimally Debulked, Advanced Endometrial Carcinoma	Stage I-IVA EC	Carboplatin + paclitaxel ± cisplatin: Arm 1: cisplatin + VDRT or BT. After chemoradiotherapy, paclitaxel + carboplatin Arm 2: paclitaxel + carboplatin	VDRT 5 days a week for 5–6 weeks or BT over 2–3 weeks. Concurrent with chemotherapy	Number of participants with recurrence, progression or death	Number of participants with acute late adverse effects, OS, patient-reported-neuropathy symptoms and QOL
NCT00807768 [Active, not recruiting]	III	601	A Phase III Trial of Pelvic Radiation Therapy Versus Vaginal Cuff Brachytherapy Followed by Paclitaxel/Carboplatin Chemotherapy in Patients With High Risk, Early Stage Endometrial Carcinoma	High risk stage I-II EC	Carboplatin + paclitaxel: Arm 1: IMRT ± BT when specified Arm 2: BT + carboplatin + paclitaxel	IRMT: 25–28 fractions during 5–6 weeks. BT: 3–5 high-dose rate treatments over 2 weeks or 1–2 low-dose rate over 1–2 days. Chemotherapy given within 3 weeks after initiating BT	Number of participants with recurrence or death events at primary analysis	Number of participants with death events and with sites of recurrence, patient-reported fatigue and neurotoxicity and QOL
NCT00492778 [recruiting]	II	164	A Randomized Trial of Pelvic Irradiation With or Without Concurrent Weekly Cisplatin in Patients With Pelvic-Only Recurrence of Carcinoma of the Uterine Corpus	Recurrent EC	Cisplatin: Arm 1: EBRT followed by intracavitary or interstitial rate interstitial BT Arm 2: EBRT + cisplatin, followed by BT	EBRT on days 1–5 for 5 weeks. Intracavitary low-dose or high-dose rate BT or low-dose rate interstitial BT. Concurrently with chemotherapy	PFS	OS, Prognostic significance of tumor size, tumor location (vaginal only vs. all others) and histology and incidence of adverse effects

Abbreviations: BT: brachytherapy; DFS: disease-free survival; EBPR: external beam pelvic radiation; EC: endometrial cancer; IMRT: Intensity-modulated radiation therapy; ORR: overall response rate; OS: overall survival; PFS: progression-free survival; QOL: quality of life; RT: radiotherapy; VDRT: volume-directed radiation therapy.

## Supplementary Materials

The following are available online at https://www.mdpi.com/2072-6694/12/7/1906/s1, Figure S1 Effects on overall survival and progression-free survival of mutations in the PTEN/PI3K/AKT pathway in endometrial cancer. Figure S2 Effects on overall survival and progression-free survival of mutations in the PTEN/PI3K/AKT pathway in endometrial cancer.
