# Supplementary material for: Determinants of Sensitivity to Radiotherapy in Endometrial Cancer
Source: Cancers (Basel). 2020 Jul 15;12(7):1906. doi: 10.3390/cancers12071906 (PMC7409033; doi:10.3390/cancers12071906)
Supplement: Supplementary file 1 [file cancers-12-01906-s001.pdf]

Supplementary Material

Methods of the survival curves (Supplementary Figure 1 and 2)

To elaborate the survival curves, *in silico* analysis using public data sets from cBioPortal for cancer genomics was performed. Four datasets from The Cancer Genome Atlas (TCGA) containing molecular and clinical data from patients with uterine corpus endometrial cancer or endometrial cancer were selected. These were: i) Endometrial cancer-MSK 2018 (197 samples), ii) TCGA Firehose Legacy (548 samples), iii) TCGA Nature 2013 (373 samples) and iv) TCGA PanCancer Atlas (529 samples). A total of 1,647 samples from 1,638 patients were queried against PTEN/PI3K/AKT pathway genes, which were: *PIK3CA*, *PIK3R1*, *PTEN*, *AKT1*, *AKT*2 and *AKT3*. The existence of mutations in the PI3K pathway is highly prevalent in the cohorts. Samples were divided into two different groups: Altered group, containing 1,122 samples with mutations on the aforementioned genes, and non-altered group, containing 515 samples without mutations in those genes (Supplementary Figure 1A). In addition, the altered group was further stratified in different subgroups depending on the specific mutated oncogene (Supplementary Figure 1B-D and Supplementary Figure 2A-C). Kaplan-Meier curves for overall survival (OS) and progression free survival (PFS) were performed based on patients' data and statistical significance between groups was evaluated using log rank test and determined when $p<0.05$.

Figure captions

Supplementary Figure 1 | Effects on overall survival and progression-free survival of mutations in the PTEN/PI3K/AKT pathway in endometrial cancer. Left: Kaplan-Meier curves of overall survival and Right: progression free survival of mutations in A. All six genes queried, B. *PTEN*, C. *PIK3CA* and D. *PIK3R1*. Statistical significance between groups was evaluated using log rank test and determined when $p<0.05$.

Supplementary Figure 2 | Effects on overall survival and progression-free survival of mutations in the PTEN/PI3K/AKT pathway in endometrial cancer. Left: Kaplan-Meier curves of overall survival and Right: progression free survival of mutations in A. *AKT1*, B. *AKT2* and C. *AKT3*. Statistical significance between groups was evaluated using log rank test and determined when $p<0.05$.

Supplementary Figure 1

A *ALL PI3K PATHWAY BIOMARKERS*

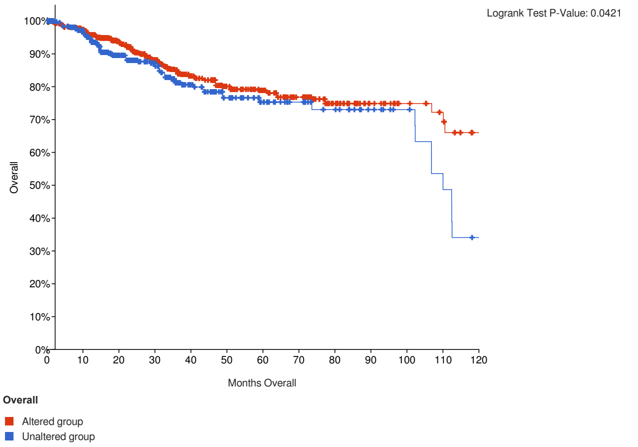

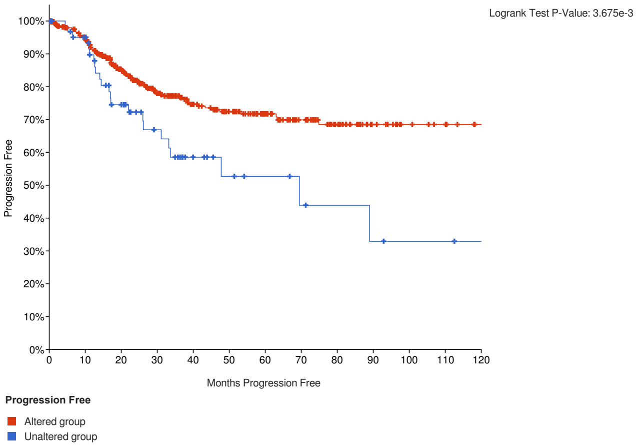

B *PTEN*

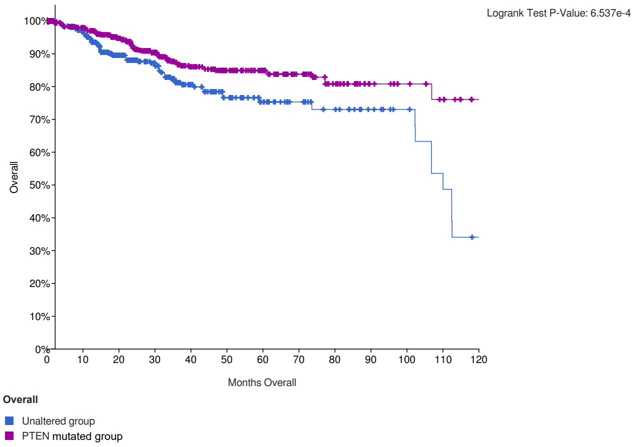

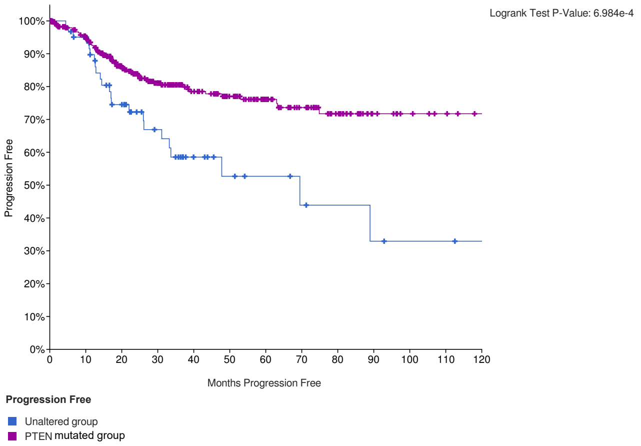

C *PIK3CA*

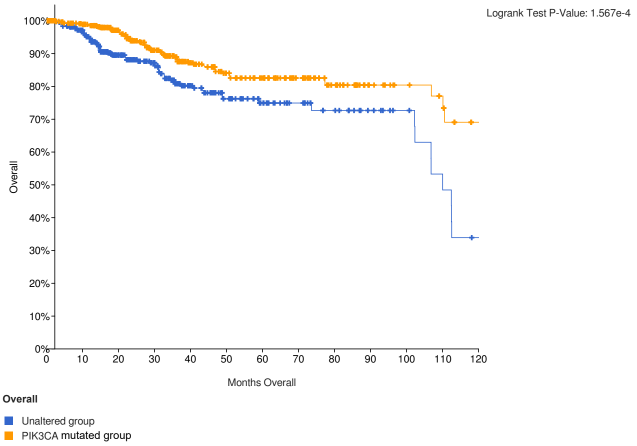

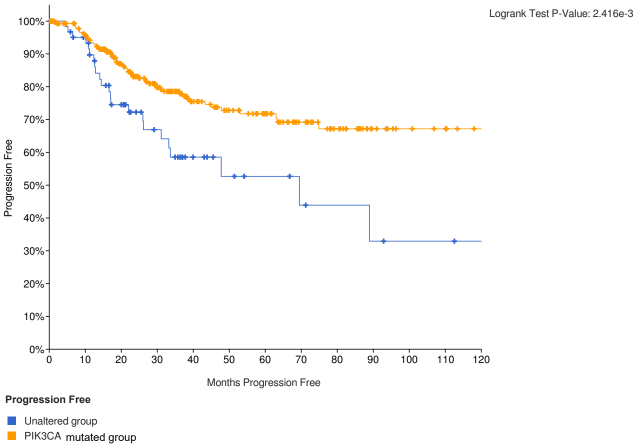

D *PIK3R1*

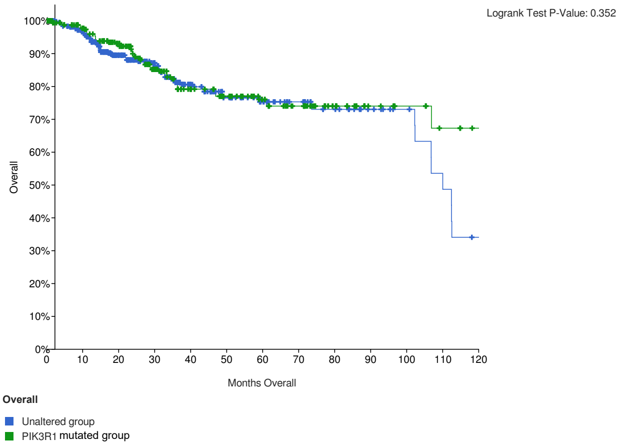

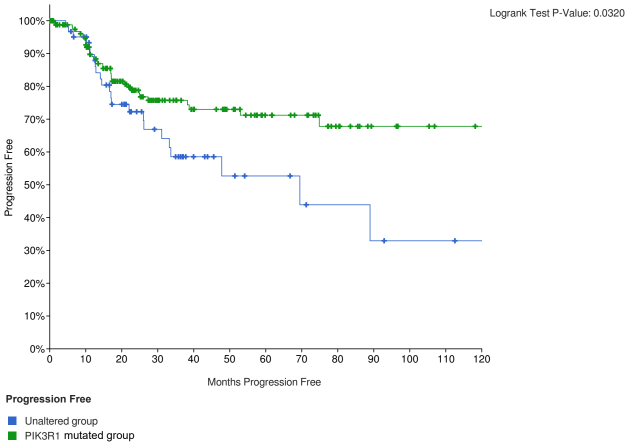

OVERALL SURVIVAL **PROGRESSION-FREE SURVIVAL**

Supplementary Figure 2

A *AKT1*

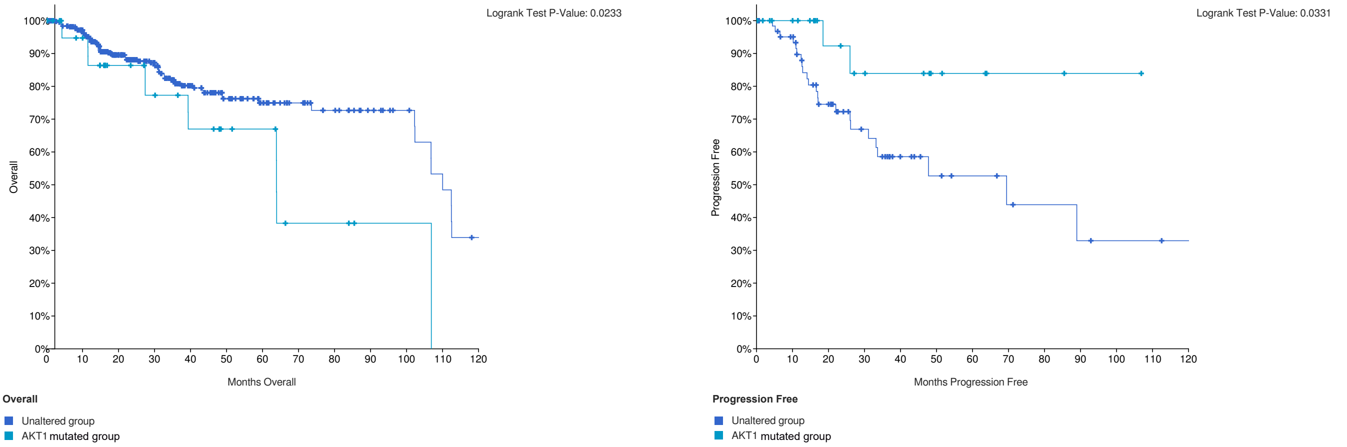

B *AKT2*

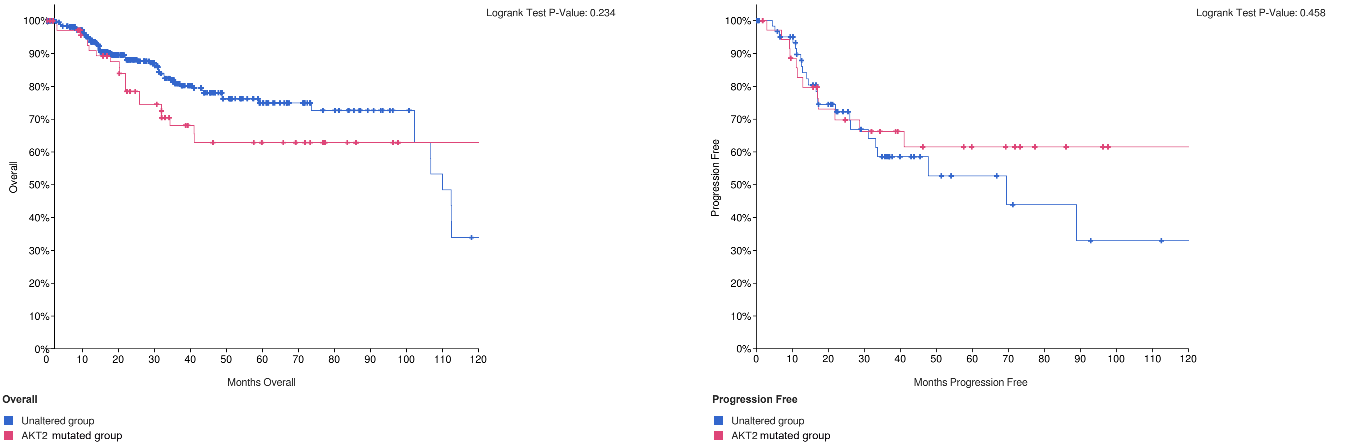

C *AKT3*

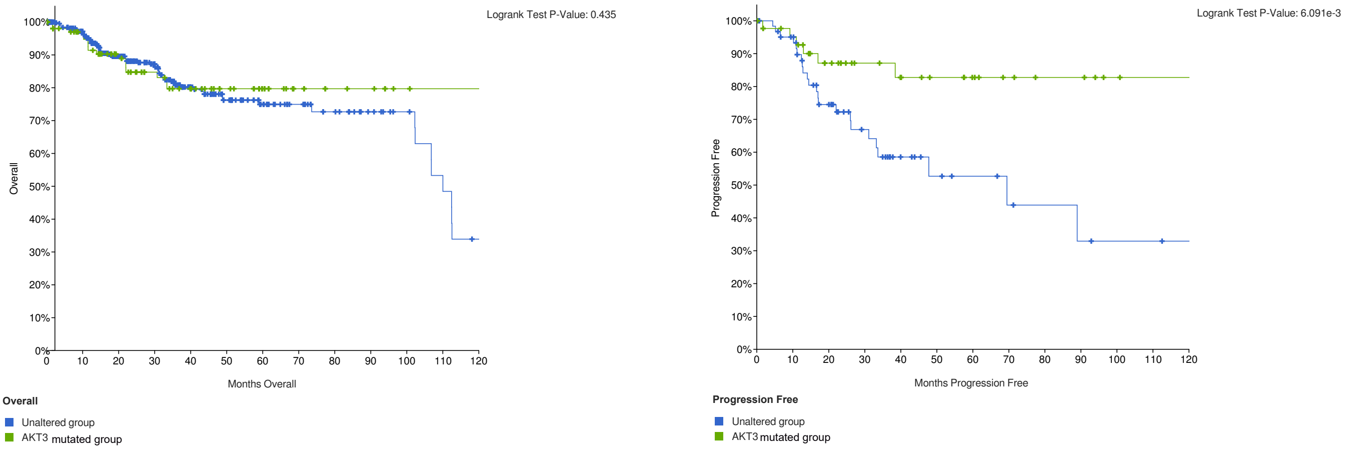

OVERALL SURVIVAL **PROGRESSION-FREE SURVIVAL**
